# Development of a Consolidated Health Facility Masterlist Using Data From Polio Electronic Surveillance in the World Health Organization African Region

**DOI:** 10.2196/54250

**Published:** 2024-06-21

**Authors:** Marie Aimee Babona Nshuti, Kebba Touray, Ticha Johnson Muluh, Godwin Akpan Ubong, Reuben Opara Ngofa, Bello Isa Mohammed, Ishimwe Roselyne, David Oviaesu, Evans Mawa Oliver Bakata, Fiona Lau, John Kipterer, Hugh Henry W Green, Vincent Seaman, Jamal A Ahmed, Modjirom Ndoutabe

**Affiliations:** 1 World Health Organization Regional Office for Africa Brazzaville Congo; 2 Bill & Melinda Gates Foundation Seattle, WA United States

**Keywords:** African region, electronic surveillance, geographic information systems, Global Polio Eradication Initiative, integrated supportive supervision, polio

## Abstract

Geospatial data reporting from surveillance and immunization efforts is a key aspect of the World Health Organization (WHO) Global Polio Eradication Initiative in Africa. These activities are coordinated through the WHO Regional Office for Africa Geographic Information Systems Centre. To ensure the accuracy of field-collected data, the WHO Regional Office for Africa Geographic Information Systems Centre has developed mobile phone apps such as electronic surveillance (eSURV) and integrated supportive supervision (ISS) geospatial data collection programs. While eSURV and ISS have played a vital role in efforts to eradicate polio and control other communicable diseases in Africa, disease surveillance efforts have been hampered by incomplete and inaccurate listings of health care sites throughout the continent. To address this shortcoming, data compiled from eSURV and ISS are being used to develop, update, and validate a Health Facility master list for the WHO African region that contains comprehensive listings of the names, locations, and types of health facilities in each member state. The WHO and Ministry of Health field officers are responsible for documenting and transmitting the relevant geospatial location information regarding health facilities and traditional medicine sites using the eSURV and ISS form; this information is then used to update the Health Facility master list and is also made available to national ministries of health to update their respective health facility lists. This consolidation of health facility information into a single registry is expected to improve disease surveillance and facilitate epidemiologic research for the Global Polio Eradication Initiative, as well as aid public health efforts directed at other diseases across the African continent. This review examines active surveillance using eSURV at the district, country, and regional levels, highlighting its role in supporting polio surveillance and immunization efforts, as well as its potential to serve as a fundamental basis for broader public health initiatives and research throughout Africa.

## Introduction

After a decades-long, global effort, wild-type polioviruses (WPV) 2 and 3—the cause of poliomyelitis (polio)—were eliminated worldwide in 2015 and 2019, respectively [1]. However, WPV1 remains endemic in Afghanistan and Pakistan, where mountainous terrain, armed conflict, and societal and political factors have impeded polio immunization and surveillance [1,2]. More than 95% of polio cases are asymptomatic or associated with only mild, cold-like symptoms [3]. However, ~1 in 200 infected individuals develop acute flaccid paralysis (AFP) and paralytic polio, which have case fatality rates of 2% to 5% among children and up to 15% to 30% among adolescents and adults [3,4].

Infected persons, including asymptomatic individuals, may shed the polio virus in nasopharyngeal secretions or stool for several days to weeks, increasing the risk of transmission of this incurable and potentially fatal disease [3]. A Pakistani lineage of WPV1 was recently found in Mozambique and Malawi, demonstrating that polio risk extends far beyond the communities where it is endemic [5]. In addition, circulating vaccine-derived poliovirus (cVDPV) may emerge in populations with low immunization rates due to the genetic instability of the live-attenuated viruses included in the oral polio vaccine [6,7]. These mutated, neurovirulent strains produce paralysis much like WPV-associated polio [7]. They also account for the vast majority of polio cases [8].

To stop the spread of polio, the World Health Organization (WHO) Global Polio Eradication Initiative (GPEI) advocates intensive efforts to identify AFP cases in children aged <15 years (ie, disease surveillance)—both routine immunization (coverage >95%) and immunization campaigns to boost vaccination coverage to >95% and enhance herd immunity—to eliminate the potential for cVDPV transmission, and the deployment of novel type 2 oral polio vaccine, which reduces transmission of cVDPV2, the most common cause of polio [2,9,10]. The complementary strategies of surveillance and immunization led to the African continent being declared indigenous WPV-free in 2020, and since GPEI efforts began in 1988, nearly 1 million polio deaths were averted [11,12]. However, disruptions in GPEI activities during the COVID-19 pandemic contributed to a resurgence in African polio cases [2]. Since 2020, >1000 polio cases have been documented, including >600 reported in Africa in 2022, which highlights the urgent and ongoing need for intensive disease surveillance and immunization to prevent the further spread of polio [8,12-14].

Geospatial data reporting from surveillance and immunization efforts is a key aspect of WHO GPEI polio-eradication efforts in Africa, which are coordinated through the WHO Regional Office for Africa (AFRO) Geographic Information Systems (GIS) Centre. To ensure the accuracy of field-collected data, the AFRO GIS Centre has developed mobile phone apps such as electronic surveillance (eSURV) and integrated supportive supervision (ISS) geospatial data collection programs. These forms, apart from reporting on unreported AFP cases, are currently being used to create a consolidated Health Facility Masterlist for the WHO AFRO, which is a repository database containing validated baseline data for all health facilities in the African region. The Health Facility Masterlist contains comprehensive listings of the names, locations, and types of health facilities in each WHO AFRO member state. Field officers are responsible for documenting and transmitting geospatial location information regarding health facilities and traditional medicine (TM) sites using the eSURV form during their visits to active surveillance sites. The functionality of these facilities, as described in the Health Facility Masterlist, goes beyond mere listing. It involves the integration of health facility data and traditional health sites to enhance disease surveillance capabilities, particularly for polio. The Masterlist is intended to serve as a dynamic resource for health workers, enabling better planning and targeting of health interventions, improving response times to disease outbreaks, and facilitating more effective deployment of resources. The consolidated Masterlist should support at the operational level health strategies and interventions by ensuring surveillance is done properly at health facilities, and by providing a comprehensive, accessible, and up-to-date database of health facilities across the WHO African region. This review delves into the use of current and comprehensive health facility lists at the district, country, and regional levels, highlighting their role in supporting polio surveillance and immunization efforts, as well as their potential to serve as a fundamental basis for broader public health initiatives and research endeavors across Africa.

## Master Facility List Program in Africa

A master facility list (MFL) is a complete, up-to-date, authoritative listing of health care facilities in any given country. Each MFL includes the information needed to accurately identify each facility, including the name, unique identifier, location, facility type, ownership, operational status, contact information for key personnel, types of services offered, and number of beds. The MFL should be validated, continuously updated, and accessible to all authorized stakeholders [15].

To date, MFLs have been updated manually. This process typically involves the country team sharing new facility information as it becomes available or through the use of the “Other” option. When a facility is not found in the existing list and is subsequently added as an “Other” option, it necessitates a manual update of the list. The upcoming version of eSURV should, however, bring significant improvements. It will incorporate a health facility registry, which will streamline the process of adding new facilities and updating existing facility forms.

Several countries have successfully compiled and made their MFLs available online, marking significant progress in health care information accessibility over the past 5 years. Notable examples include Zambia [16], Botswana [17], and Kenya [18]. There are also broader initiatives such as the WHO AFRO Integrated African Health Observatory MFL database and the Geolocated Health Facilities Data Initiative, aimed at enhancing health care planning and delivery with a more comprehensive MFL.

Despite these advancements, challenges remain in ensuring the continuous updating and maintenance of these lists. While eSURV visits are conducted regularly at health facilities—including traditional sites such as those of traditional practitioners—to verify and augment existing MFLs with new or missing facilities, the objective of sustaining up-to-date MFLs relies heavily on the diligent efforts of field officers conducting active surveillance. This ongoing process is crucial for maintaining the accuracy and utility of MFLs over time.

## MFL Resource Package and Training

Before the availability of electronic databases, African MFLs were beset by challenges common to all paper-based data management systems, including out-of-date information, omissions, inconsistencies, and inaccuracies [19]. In addition, updating or correcting paper-based files and sharing the information with stakeholders such as other government ministries, public health agencies, and charitable organizations could be slow and resource-intensive. To address these shortcomings, the WHO and the United States Agency for International Development developed a set of guidelines for the implementation of a software-based facility registry service that makes the MFL accessible to stakeholders such as insurance companies; charitable groups undertaking health interventions; researchers assessing health system performance; and personnel involved in health management information systems, disease surveillance, and supply chain management [15,20]. A training program was also developed to help country teams establish or improve their MFLs. The target audience for this program includes ministry officials, facility list managers, data curators, national and regional health ministry personnel, and staff from nongovernmental organizations. Participants are trained in the purpose and contents of an MFL, MFL assessment, governance, data content, incorporation of geographic coordinates, establishment of the data set and registry service, ongoing maintenance, and sharing of the MFL, with a final session devoted to crafting an action plan for MFL creation and implementation.

## TM Sites in MFLs

An important component of modern MFL development is the inclusion of TM sites in the database. Traditional healers serve as first-line health care providers for most of the population in Africa, where there is only 1 medical physician for every 40,000 people, compared with a 1:500 ratio of traditional healers to African residents [21-23]. Although some TM practices are associated with misdiagnoses leading to missed cases and ineffective treatment, traditional healers can have a strong, positive impact on the health and wellness of the people they serve when their products and practice are properly regulated and integrated into health systems [22]. These practitioners are also an important resource for disease surveillance and immunization efforts and may be trained to conduct community disease surveillance in areas where there is a shortage of medical facilities and formally trained health care providers.

## eSURV and ISS in Practice

### About eSURV

eSURV was developed by the WHO AFRO GIS Centre as an electronic health solution to enable surveillance agents to use mobile phones to document active case searches for polio and other infectious disease cases in health facilities and the community. The app comprises an electronic form based on a checklist that records the geographic location of health facilities visited by data collectors based on the global positioning system software on the user’s phone. The tool is used in 46 WHO AFRO member states, where case search activity is automatically recorded on country-specific modules on the GIS servers. The accumulated data are used to identify outbreaks of not only polio, but also other priority diseases such as COVID-19 [24,25]. As a result of data being continuously transmitted to GIS servers in near real time, areas with little or no surveillance activity can be promptly identified and corrective actions can be taken.

### About ISS

**Figure 1:**
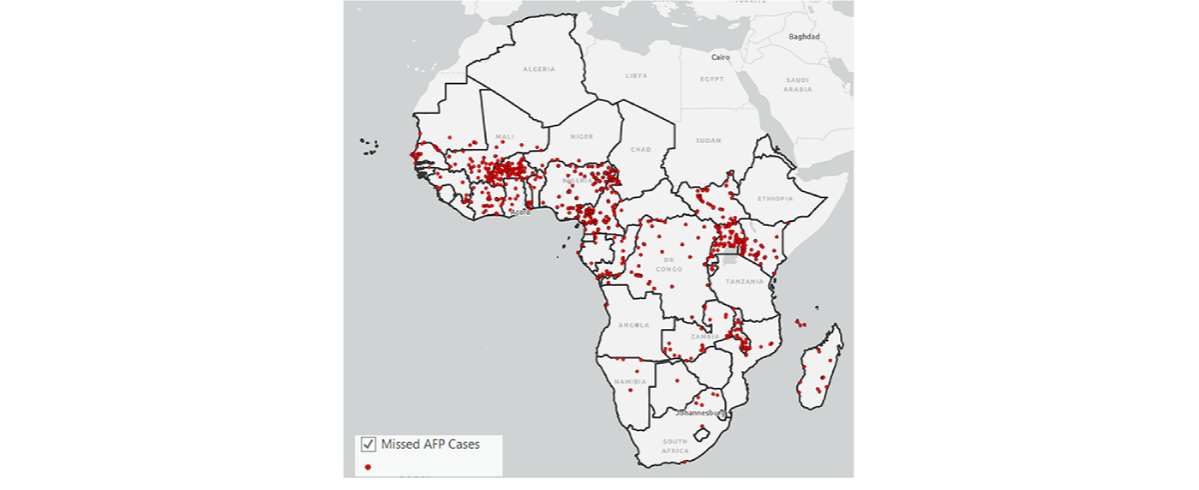
Number of districts with unreported cases of AFP in 2021 as revealed through the use of integrated supportive supervision. AFP: acute flaccid paralysis.

Supportive supervision fosters high-quality disease surveillance and immunization best practices through systematic visits to priority sites (health facilities and surveillance sites, including TM practitioner sites) by supervisory WHO and national health ministry personnel, where they conduct monitoring, evaluation, and on-the-job training of health workers. To document these activities, the supervisors use ISS, a mobile phone app consisting of an integrated electronic checklist used for the supervision of active AFP case finding and routine polio immunization [24]. Approximately 40% of checklist questions focus on case surveillance for AFP and other vaccine-preventable diseases, whereas the other 60% assess routine immunization practices. Through ISS, supervisors promote understanding and recognition of AFP among frontline workers and help them keep immunization records up to date [26]. In addition, ISS facilitates the detection of previously missed AFP cases (Figure 1 and Table 1).

**Figure 2:**
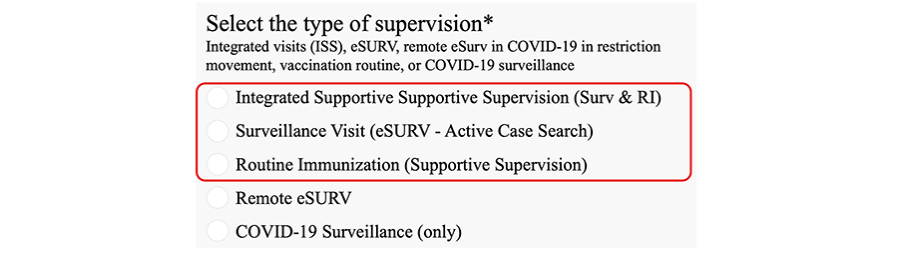
Screenshot of ISS. eSURV: electronic surveillance; ISS: integrated supportive supervision; RI: routine immunization; Surv: surveillance.

To ensure uniformity and standardization, the ISS tool includes 4 question categories the supervisor can ask frontline workers. The ISS checklist includes questions about both AFP cases and polio vaccinations. The app also includes the eSURV checklist, consisting of questions only about active AFP cases. A third category covers only routine polio immunizations and a fourth focuses on COVID-19 case surveillance (Figure 2 and Table 2).

**Table 1:** Number of districts with unreported cases of acute flaccid paralysis in 2021 as revealed through the use of integrated supportive supervision.

District	Cases, n
Cameroon	350
Burkina Faso	198
Congo	103
Kenya	102
South Sudan	102
Democratic Republic of Congo	94
Mali	70
Uganda	64
Côte d’Ivoire	46
Malawi	42
Senegal	30
Togo	30
Zambia	28
Niger	18
Madagascar	17
Gabon	11
Chad	10
Ghana	10
Mauritania	9
Guinea	8
Namibia	8
South Africa	8
Comoros	7
Central African Republic	5
Benin	4
Liberia	4

**Table 2:** Frequency of type of supervision in Cameroon and Mali from July 2020 through June 2021.

Type of supervision	Cameroon cases, n^a^	Mali cases, n^b^
Integrated supportive supervision (surveillance and routine immunization)	13,152	3099
Surveillance visit (eSURV^c^—active case search)	8171	4811
Routine immunization (supportive supervision)	545	213
Remote eSURV	125	34
COVID-19 surveillance (only)	335	60

^a^Displaying 22,328 of 63,343 records.

^b^Displaying 8217 of 20,639 records.

^c^eSURV: electronic surveillance.

## Frequency of eSURV and ISS Visits to Health Facilities

The WHO recommends regular surveillance and supervisory visits to health facilities, with the frequency of visits being dependent on the priority level of the site (Table 3). The guidance suggests weekly visits to high-priority sites, including 3 eSURV and 1 ISS visit/mo, a visit every 2 weeks (1 eSURV and 1 ISS visit/mo) to medium-priority sites, and 1 ISS visit/mo to low-priority sites.

**Figure 3:**
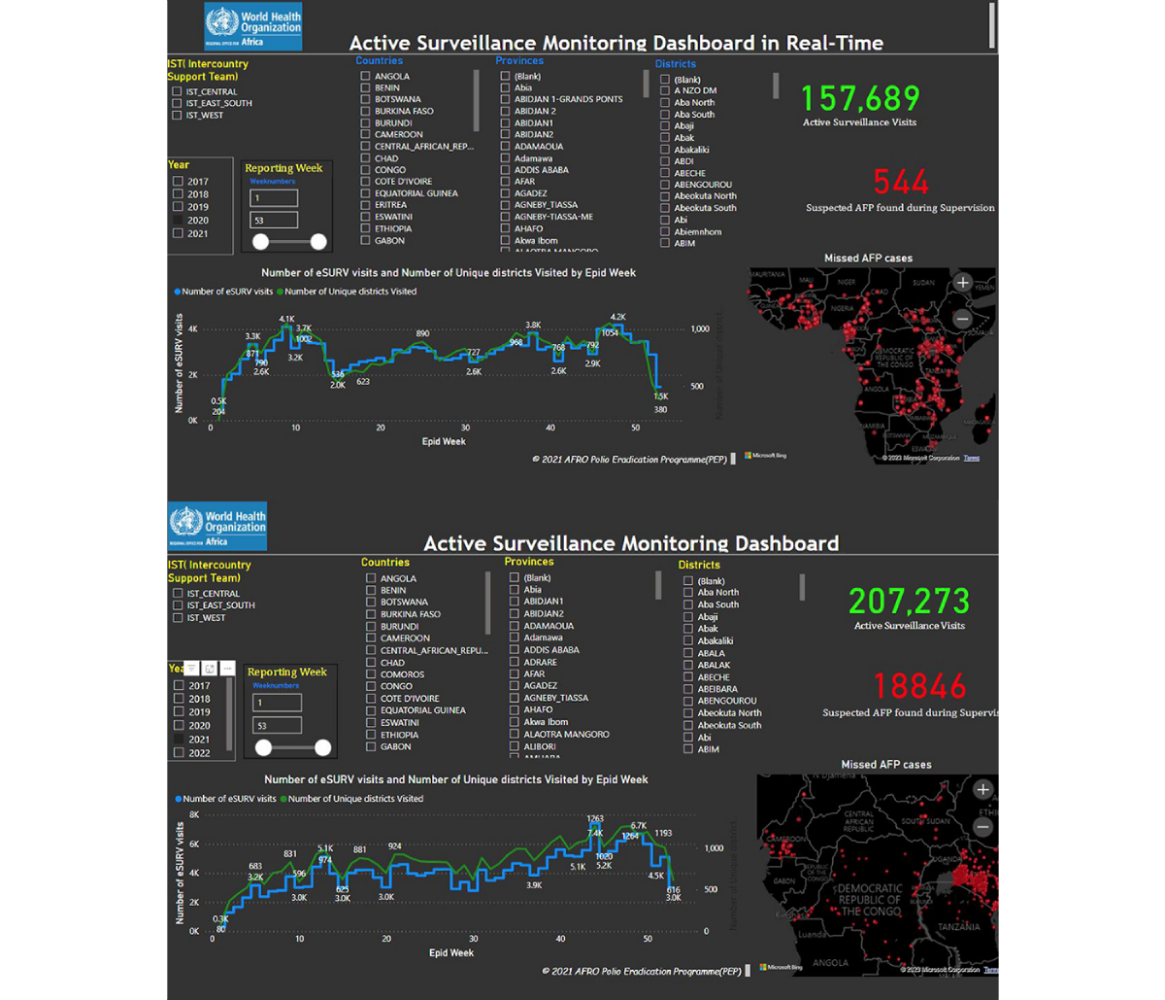
Documented active surveillance in 2020 (top panel) and 2021 (bottom panel). AFP: acute flaccid paralysis; AFRO: Regional Office for Africa; eSURV: electronic surveillance; IST: Intercountry Support Team; PEP: Polio Eradication Programme.

In the past, the numbers of actual eSURV and ISS visits have been reported to be lower than recommended, even in countries where ISS visits are required for regional certification [26]. Adherence to visit frequency recommendations appears, however, to be improving. Between 2020 and 2021, active eSURV visits increased by ~25,000, which permitted the identification of ~4000 missed AFP cases (Figure 3).

**Table 3:** Active surveillance site priority level and recommended visit frequency.

Site priority	Target/wk				Target/mo	
	Wk 1	Wk 2	Wk 3	Wk 4	Completed	Partial
Highest: structure or person located in a high-risk area (eg, refugee or IDP^a^ camp)	2	2	2	2	8/mo: weekly target 100% met	4/mo: ≥2 wk, 50% completed
High: structure or person from which an AFP^b^ case would very likely seek care	1	1	1	1	4/mo: weekly target 100% met	2/mo: ≥2 wk, 50% completed
Medium: structure or person from which an AFP case would likely seek care	1	0	1	0	2/mo: have been visited once every ≥2 wk	1/mo: ≥1 wk, 50% completed
Low: structure or person from which an AFP case would perhaps seek care	0	0	0	1	1/mo: visited ≥1 wk	—^c^

^a^IDP: internally displaced person.

^b^AFP: acute flaccid paralysis.

^c^Not available.

## Geospatial Data Entry

When a field officer visits a health facility, the geolocation data are automatically transmitted to that country’s eSURV and ISS database. Field-collected data are synchronized on the server, and the dashboard is viewable by WHO and country health ministry personnel.

**Figure 4:**
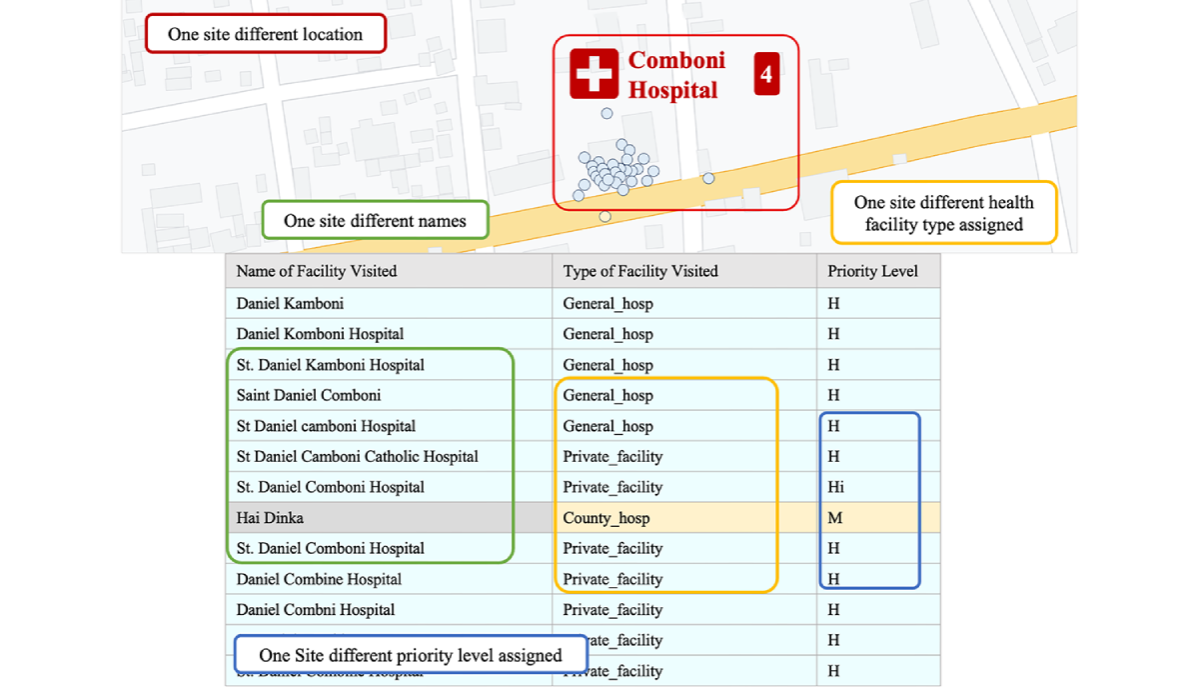
Active surveillance data from 2022 showing a single site with multiple names in the master facility list database.

One of the challenges is that a single site may have multiple names in the database, resulting in site identification mismatches or multiple sites recorded for the same geolocation (Figure 4). To address this problem, eSURV includes data entry constraints, with a dropdown menu of the country’s MFL from the health observatory facility list or, directly, an inventory of the health facility list from the country team, which helps prevent data entry and server synchronization errors [19]. To allow for the entry of previously unrecorded sites or those missing from the list, the program includes an “Other” option, whereby data collectors can enter new data.

## Contribution of eSURV and ISS to Polio Eradication Efforts

The eSURV and ISS apps provide real-time georeferenced surveillance and immunization data that can be accessed by polio program staff and used to audit data and evaluate data collector performance and accountability. In the past, paper-based systems were found to be not only slow, but also susceptible to data fabrication and omissions [19]. In contrast, real-time data transmission with mobile phone apps increases surveillance sensitivity and transparent data management increases WHO GPEI confidence in program results. The Open Data Kit (ODK) app currently faces some compatibility issues with iOS users. A few mechanisms have, however, been implemented to facilitate access for iPhone users. An example of this is the introduction of GIC Collect, which is compatible with iOS, thereby addressing some of these constraints. GIC Collect works as a port for the ODK for use with an iPhone.

**Figure 5:**
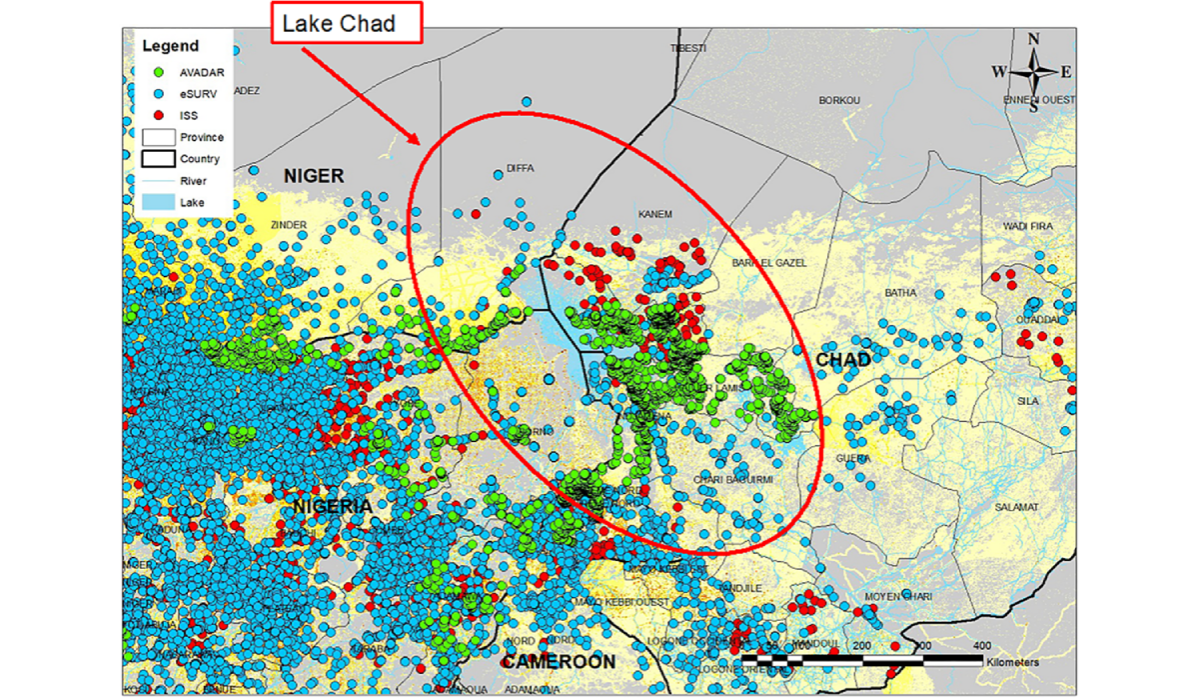
Closing surveillance gaps in the Lake Chad area in 2019. Use of mobile eSURV and ISS, along with AVADAR for geospatial documentation of surveillance of acute flaccid paralysis and polio immunizations among nomadic populations living in the Lake Chad region of Africa. AVADAR: Auto Visual Acute Flaccid Paralysis Detection and Reporting; eSURV: electronic surveillance; ISS: integrated supportive supervision.

The data provided through eSURV and ISS contributed to the attainment of WPV-free certification for the WHO African region [24]. For example, these tools were essential in AFP surveillance and immunization of >40,000 children of nomads living in 62 high-risk districts in and around the remote Lake Chad region (Figure 5) [25]. The eSURV and ISS data facilitated the identification and location of at-risk groups, who continue to be monitored for AFP cases [27].

Multiple studies have documented improvements in African polio public health interventions after the implementation of eSURV and ISS [19,26,28-32]. A preliminary analysis of the transition from a paper-based system to eSURV showed that the average case-reporting time in Africa decreased by 90% from a mean of 87.6 to 8.7 hours [32]. The time from case identification to entry into a polio case tracking database was reduced by 72% from a mean of 30 to 8 days. Another analysis by the same author group showed that the completeness of data collected via eSURV and ISS was >95% across 5 countries in sub-Saharan Africa, with error rates that ranged from 0.01% to 0.03%. The authors attributed the low error and high completion rates to inherent aspects of the software, including data entry constraints, skip logic, and enforced validation, as well as real-time data sharing. The latter enabled supervision and monitoring of the flow of data that permitted supervisors to detect data fabrication and omissions, which have been documented with paper-based systems [19].

In another study, the implementation of ISS in Zambia improved the updating of immunization records in 7 of 9 provinces studied, with up to a 53% increase in the proportion of health facilities with updated immunization records [26]. In addition, ISS had a statistically significant, positive impact on knowledge of AFP case definition and documentation of AFP case files.

## Development of a Consolidated Health Facility Masterlist—Integration of eSURV and ISS With MFLs

Despite improvements in the frequency and accuracy of data collection with eSURV and ISS, several challenges have been identified. First, the total number of health facilities and TM sites in Africa has not been fully documented. The lack of a ddenominator of known sites limits the ministry of health and WHO personnel’s ability to assess the completeness of surveillance coverage. In addition, the lack of a validation mechanism for the reference list of health facilities has the potential to contribute to database errors. To address these gaps, the consolidated Health Facility Masterlist was developed and is managed by the WHO AFRO GIS Centre in close cooperation with the respective health ministries of each member state.

The Health Facility Masterlist uses existing MFLs provided by each country as references, which are compared with health facility lists compiled from eSURV and ISS data. In a structured process that is still ongoing, WHO AFRO surveillance and GIS personnel cross-reference eSURV and ISS lists from each country against that country’s MFL and consolidate the information for health facilities (including TM sites) with ≥95% similarity. Facilities with <95% similarity are returned to each country, where a designated focal person validates the site information using a health facility validation checklist web app that was developed for Masterlist consolidation and validation. The validated facility uses a standard operating procedure, developed at the operational level by the AFRO GIS Centre and validated by the country surveillance and data management team, to agree on the nomenclatures of a health facility name and health facility type as they differ from one country to another. Validated facilities are then added to the consolidated Health Facility registry form as updates. As data collectors discover and record new facilities (eg, previously unrecognized TM sites) using the Site Registry form, the in-country focal person validates the facilities using the MFL validation checklist. After verification by the in-country teams (ministry of health and WHO), the new facilities are added to the consolidated health facility registry. The AFRO GIS Centre, in collaboration with the surveillance team at the country level, conducts updates on a priority level twice yearly. The Masterlist includes the following variables: site name, location based on administrative boundary, geocoordinates, priority level, type of site, level of care, operating status, vaccination status, ownership, and auto-generated unique identifier.

**Figure 6:**
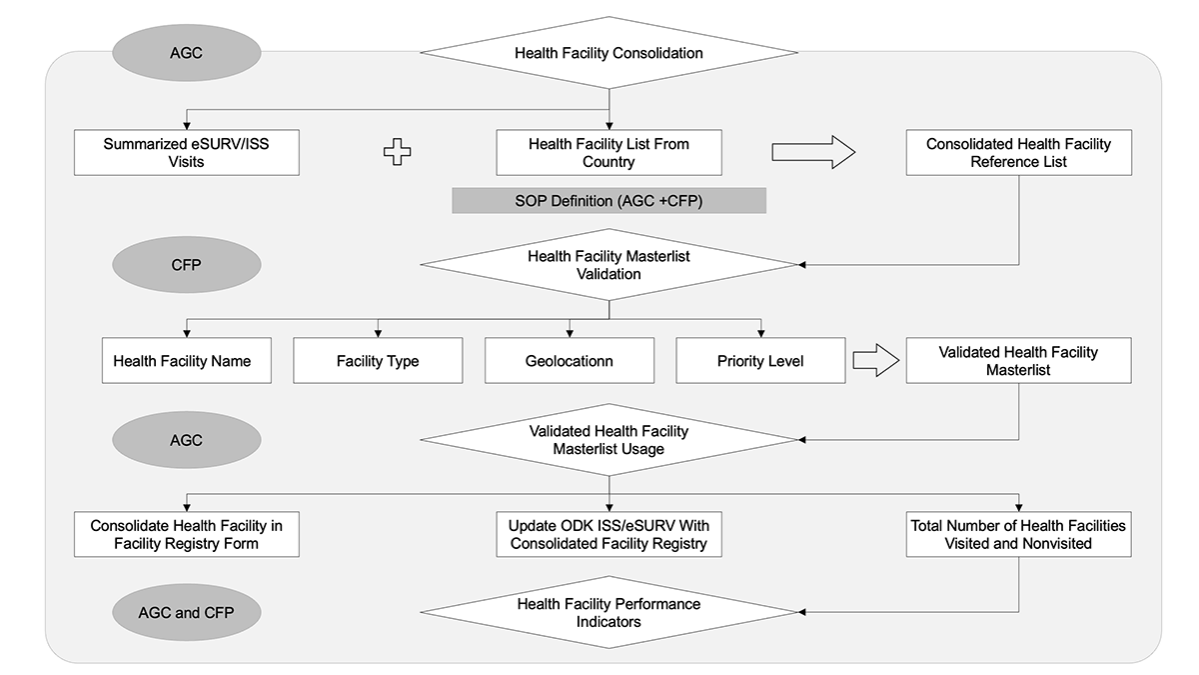
Schematic depiction of the development of consolidated Health Facility Masterlist. AGC: World Health Organization Regional Office for Africa Geographic Information Systems Centre; CFP: Country Focal Point; eSURV: electronic surveillance; ISS: integrated supportive supervision; ODK: Open Data Kit; SOP: standard operating procedure.

Once the consolidated Health Facility Masterlist is complete, the data can be used by the WHO AFRO to more accurately evaluate the completeness of surveillance and immunization efforts, as well as assess the performance of health facilities (Figure 6).

The Health Facility Masterlist is specifically designed for use at the operational level. Any digital surveillance or data collection activities carried out at the health facility level can make use of the health facility registry as a valuable resource. The primary objective is to continually update and enhance the registry, aiming to benefit all countries in the WHO African region. Additionally, the registry will be maintained as a web service, enabling automated integration with various forms such as eSURV, vaccine management, and other ODK forms that are administered at the health facility level, facilitating a comprehensive and streamlined approach. The primary limitation of the use of the Health Facility Masterlist lies in elucidating the core method for updating the health facility process after consolidation. At present, the AFRO GIS Centre is working on a well-defined system commonly called the “eSURV Companion App” for maintaining, consolidating, and validating health facility data with eSURV. This issue will necessitate further examination in subsequent research related to this paper.

To prevent stagnation in consolidating the Health Facility Masterlist, the primary strategy is to uphold a robust active surveillance system through the use of eSURV, which is currently used by 46 countries in the WHO African region for reporting active surveillance visits. The exclusion of even one country, however, remains a significant hurdle in achieving full consolidation. Furthermore, a key recommendation is to encourage the adoption of the latest eSURV Companion App by 2024, ensuring comprehensive maintenance of the consolidated Health Facility Masterlist over time.

## Conclusions

The eSURV and ISS mobile health solutions have played a vital role in efforts to eradicate polio and control other communicable diseases in Africa. Data compiled from eSURV and ISS over the last 5 years are being used to develop, update, and validate MFLs throughout Africa. Moreover, the ongoing consolidation of health facility information into a single health facility registry that covers the WHO African region should further improve disease surveillance and facilitate epidemiologic research. These efforts will support not only polio eradication in Africa, but also public health efforts directed at other diseases across the continent.

## Abbreviations

AFP: acute flaccid paralysis
AFRO: Regional Office for Africa
cVDPV: circulating vaccine-derived poliovirus
eSURV: electronic surveillance
GIS: Geographic Information System
GPEI: Global Polio Eradication Initiative
ISS: integrated supportive supervision
MFL: master facility list
ODK: Open Data Kit
TM: traditional medicine
WHO: World Health Organization
WPV: wild-type poliovirus

## Data Availability

The data that support the findings of this study are openly available from the WHO AFRO GIS Centre at the Rapid Response Team Hub [33]. The authors confirm that the data supporting the findings of this study are available within this paper.
